# Infection of Endothelial Cells by Dengue Virus Induces ROS Production by Different Sources Affecting Virus Replication, Cellular Activation, Death and Vascular Permeability

**DOI:** 10.3389/fimmu.2022.810376

**Published:** 2022-02-02

**Authors:** Lana Monteiro Meuren, Elisa Beatriz Prestes, Michelle Premazzi Papa, Luiza Rachel Pinheiro de Carvalho, Yasmin Mucunã Mustafá, Leandro Silva da Costa, Andrea T. Da Poian, Marcelo Torres Bozza, Luciana Barros Arruda

**Affiliations:** ^1^ Departamento de Virologia, Instituto de Microbiologia Paulo de Góes, Universidade Federal do Rio de Janeiro, Rio de Janeiro, Brazil; ^2^ Laboratório de Inflamação e Imunidade, Departamento de Imunologia, Instituto de Microbiologia Paulo de Góes, Universidade Federal do Rio de Janeiro, Rio de Janeiro, Brazil; ^3^ Department of Microbiology, Immunology and Tropical Medicine, The George Washington University, Washington, DC, United States; ^4^ Instituto de Bioquímica Médica Leopoldo de Meis, Universidade Federal do Rio de Janeiro, Rio de Janeiro, Brazil

**Keywords:** dengue, human brain microvascular endothelial cells (HBMEC), reactive oxygen species, mitochondria, NADPH oxidase, cytokines, cell death

## Abstract

Exacerbated inflammatory response and altered vascular function are hallmarks of dengue disease. Reactive oxygen species (ROS) production has been associated to endothelial barrier disturbance and microvascular alteration in distinct pathological conditions. Increased ROS has been reported in *in vitro* models of dengue virus (DENV) infection, but its impact for endothelial cell physiology had not been fully investigated. Our group had previously demonstrated that infection of human brain microvascular endothelial cells (HBMEC) with DENV results in the activation of RNA sensors and production of proinflammatory cytokines, which culminate in cell death and endothelial permeability. Here, we evaluated the role of mitochondrial function and NADPH oxidase (NOX) activation for ROS generation in HBMEC infected by DENV and investigated whether altered cellular physiology could be a consequence of virus-induced oxidative stress. DENV-infected HBMECs showed a decrease in the maximal respiratory capacity and altered membrane potential, indicating functional mitochondrial alteration, what might be related to mtROS production. Indeed, mtROS was detected at later time points after infection. Specific inhibition of mtROS diminished virus replication, cell death, and endothelial permeability, but did not affect cytokine production. On the other hand, inhibition of NOX-associated ROS production decreased virus replication and cell death, as well as the secretion of inflammatory cytokines, including IL-6, IL-8, and CCL5. These results demonstrated that DENV replication in endothelial cells induces ROS production by different pathways, which impacts biological functions that might be relevant for dengue pathogenesis. Those data also indicate oxidative stress events as relevant therapeutical targets to avoid vascular permeability, inflammation, and neuroinvasion during DENV infection.

## Introduction

Dengue virus (DENV) infection is a major public health problem worldwide, mostly in tropical and subtropical countries, affecting about 400 million people every year (1). Infection by any of the four described serotypes can induce a range of clinical manifestations, from mild to severe hemorrhagic forms, that can be fatal (2). Vascular alterations, including vasodilation and increased permeability, are major consequences of DENV infection, contributing to plasma extravasation to the tissues, hemorrhagic manifestations, and hypotension, which are hallmarks of severe disease, but may also happen at lower levels in mild and moderate disease (3, 4). Previous studies indicated that vascular disturbances result from systemic inflammation and endothelial lesion (4–7). In addition, direct infection of endothelial cells was demonstrated in histopathological analysis of different tissues from fatal cases, as well as in primary cells and different cell lines (7–11).

We have previously demonstrated that human brain microvascular endothelial cells (HBMECs) are permissive to DENV, and virus replication triggers the activation of RNA sensors, inducing the production of inflammatory cytokines, chemokines and type I interferon (9). Evidence of virus-induced cell death was also detected, but the involved mechanisms had not been addressed (9, 12). A number of studies have been unraveling the complex connections between virus sensing, cellular stress response and cell death [rev in (13)]. Some of those signals converge to the production of nitrogen and oxygen reactive species, which accumulation stimulate the secretion of inflammatory mediators, and trigger autophagy, apoptosis and necroptosis in different models of viral infections (14–18). Oxidative stress may be particularly deleterious in the vascular context, since ROS-mediated endothelial cell activation and death will contribute to vascular permeability and barrier disruption, as demonstrated under several pathological conditions (19–21). Although it has been largely demonstrated that excessive ROS impair microvessel integrity and that leukocyte-derived ROS might be an important contributor to endothelial barrier damage [rev in (21)], the role of endogenous endothelial cell-derived ROS has been poorly addressed, especially in the context of virus infections.

Increased ROS production was evidenced in *in vitro* and *in vivo* models of dengue infection. *In vitro* infection of human monocyte derived dendritic cells (Mo-DC) induced the activation of NADPH oxidases (NOX) and accumulation of intracellular ROS, which contribute to enhanced production of inflammatory cytokines and chemokines (14). NOX-derived ROS was also associated to vascular damage in a mouse experimental model, in which depletion of p47phox significantly reduced DENV-induced systemic hemorrhage, in comparison to control mice (22).

Mitochondrial oxidative phosphorylation metabolism is another major source of intracellular ROS (23, 24). However, despite previous observations of altered mitochondrial bioenergetics and morphology in *in vitro* experimental models of DENV infection, the impact of virus replication in the generation of mitochondrial-derived ROS (mtROS) had not been clearly addressed (25, 26). In fact, cellular ROS may be generated by different enzymes present in distinct intracellular sites, also including xanthan oxygenase, cyclooxygenase and lipooxygenase, further contributing to overall oxidative stress (13, 19, 27).

Here, we evaluated whether infection of HBMEC with DENV resulted in enhanced cellular ROS by NOX and mitochondrial-derived pathways and assessed the relative role of each ROS source for viral replication, endothelial activation, cell death and endothelium permeability. We observed that DENV replication triggered mitochondrial and NOX-mediated ROS production, which were essential for viral replication and cell death. Mitochondrial-derived ROS was a major inducer of HBMEC permeability, whereas NOX-derived ROS played a relevant role for endothelial activation and production of inflammatory mediators. This study reveals new information about the impact of oxidative stress caused by DENV during infection of endothelial cells and provides a new perspective for the use of antioxidants for dengue treatment.

## Material and Methods

### Cells and Virus

Human brain microvascular endothelial cell line (HBMEC) (28) was kindly given by Dr. Dennis J. Grab (The Johns Hopkins University, MD, USA). The cells were cultivated in medium M199 (M199), supplemented with 10% fetal bovine serum (FBS) (Thermo Fisher Scientific Inc), at 37°C. *Aedes albopictus* clone C6/36 cell line (ATCC^®^ CRL-1660^™^) was cultured in Leibovitz (L-15) medium (Invitrogen), supplemented with 10% FCS, tryptose phosphate (2.95 g/L), 0.75% sodium bicarbonate, and 0.2% of L-glutamine (Sigma-Aldrich), at 28°C. Baby hamster kidney cells [BHK-21 (C-13]) ATCC^®^ CCL-10^™^] were cultured in Minimum Essential Medium Eagle - Alpha Modification (α-MEM) (Thermo Fisher Scientific Inc) supplemented with 5% of FBS, at 37°C.

DENV serotype 2 strain 16681 was propagated in C6/36 cells. The supernatants of infected cells were harvested, filtered, and stored at -80°C, and virus stock was titrated by plaque assay using BHK cells, as described below. Supernatants obtained from noninfected C6/36 cells cultured under the same conditions were used as mock control. Virus inactivation was performed by 2 hours UV exposure and confirmed by qRT-PCR in HBMECs.

### Ethical Statements and Obtention of Human Primary Macrophages

Blood samples (buffy coat) were obtained from the Hemotherapy Service at the Hospital Universitário Clementino Fraga Filho (HUCFF) of Universidade Federal do Rio de Janeiro (UFRJ). The study protocol was approved by the Experimental Ethics Committee of UFRJ (Permit Number: CAAE 27600314.7.0000.5275). Fresh peripheral blood mononuclear cells (PBMCs) were obtained by Ficoll-Hypaque density gradient centrifugation and cultured for 7-10 days with RPMI medium, supplemented with L-glutamine, and 2% human serum (Thermo Fisher Scientific Inc). Macrophage differentiation was confirmed by CD68 staining and flow cytometry analysis.

### Infection of HBMECs and Macrophages

HBMECs were incubated with DENV-2 virus, with a MOI of 1, for 1h at 37°C in 5% CO_2_ atmosphere. As a control, cells were mock-treated or incubated with UV-inactivated DENV-2 (iDENV). After the adsorption, the inoculum was removed, the cells were washed with phosphate buffer saline (PBS 1x) and maintained in culture medium with 10% FBS at 37°C at 5% de CO_2_ for different periods of time (24hpi-72hpi). Primary macrophages were infected, under the same conditions, using a MOI of 2. In some experiments, the following ROS inhibitors/scavengers were added to the cultures: N-Acetylcysteine (NAC; 1mM; Merck Millipore; Darmstadt, Germany), Apocynin (Apo; 1mM; Merck Millipore); mitoTEMPO (MitoT; 50µM; Enzo Life Sciences). The culture supernatants were harvested, and the titer of infectious particles released was evaluated by plaque assay.

### Virus Titration by Plaque Assay

Titration of virus stocks and measurement of infectious particles released in the supernatants of experimental cultures were performed by plaque assay using BHK cells, as described (29). Briefly, the cells were inoculated with serial dilutions of the infected samples for 2h, at 37^0^C for virus adsorption. Then, medium was replaced with 1% carboxymethylcellulose (CMC) diluted in α-MEM medium with 1% FBS. After 5 days of culture, the cells were fixed with 1ml of 10% formaldehyde for 1h and stained with 4% crystal violet solution. Virus titers were indicated as PFU/ml.

### Analysis of Virus Replication by RT-qPCR

HBMECs and macrophages were infected with DENV-2, in the presence or absence of N-acetylcysteine. After 48hpi, cell lysates and supernatants were harvested and RNA was isolated using TRIZOL reagent (Life Technologies), according to the manufacturer’s instructions. First strand cDNA was synthesized using 2 µg RNA using High-Capacity cDNA Archive Kit (Life Technologies), according to the manufacturer’s instructions. Quantitative real-time PCR was performed using a StepOnePlus Real-time PCR system (Life Technologies) and Taqman Master Mix Reagents (Life Technologies), as described before (9).

### ROS Quantification by Flow Cytometry and Immunofluorescence

To measure total or mtROS, the cells were incubated with the probes CM-H2DCFDA (1µM/1x10^6^ cells; Thermo Fisher Scientific Inc) or MitoSOX (1µM; Thermo Fisher Scientific Inc), respectively, at different time points post infection. The cells were analyzed by flow cytometry using the FACScalibur and FlowJo software (LCC, Ashland, USA). The same methodology was used to measure ROS by fluorescence microscopy and the cells were analyzed using OLYMPUS IX81 microscopy.

### Analysis of Oxygen Consumption and Assessment of Mitochondrial Bioenergetics

Oxygen consumption rate (OCR) by control or DENV-2-infected cells was evaluated by high resolution respirometry. HBMECs (2 x 10^6^ cells) were mock-treated or infected with DENV-2 for different periods of time. Respirometry was monitored in real time using Oroboros equipment (Oxygraph-2K, Instruments, Innsbruck, Austria) and sequentially adding pharmacological inhibitors of the oxidative phosphorylation. Oligomycin (200µg/mL) was used to determine oxygen consumption not associated with ATP synthase, and the mitochondrial oxidative phosphorylation uncoupler carbonyl cyanide p-trifluoromethoxyphenylhydrazone (FCCP) (1mM) was used to allow maximum electron flow in the electron transport chain. These parameters allowed the evaluation of basal respiration (before oligomycin addition), OCR due to proton leak (uncoupled OCR; after oligomycin addition), OCR associated to ATP synthase (coupled OCR - difference between basal and oligomycin OCR), maximum respiratory capacity (after addition of FCCP), and reserve capacity (difference between FCCP and basal OCR).

### Evaluation of Mitochondrial Membrane Potential (Δψ_m_)

Mitochondrial membrane potential in mock-treated or DENV-2-infected HBMEC was evaluated using JC-1 dye (5,5′,6,6′-tetrachloro-1,1′,3,3′-tetraethylbenzimidazolyl-carbocya-nine iodide; Molecular Probes). Cells were incubated with 5μg/mL JC-1 at 37°C for 1 h. Culture medium and 10 minutes incubation with FCCP were used as negative and positive control, respectively. The cells were analyzed by flow cytometry using the FACScalibur equipment and FlowJo software (LCC, Ashland, USA), and the ratio red/green fluorescence was used to measure the membrane potential.

### Cell Viability Assays

HBMECs and macrophages were mock-treated or infected with DENV-2, in the presence or absence of ROS inhibitors. After 48hpi, macrophage metabolic activity was addressed by MTT assay, according to the manufacturers protocol (Thermo Fisher Scientific Inc). In addition, after 48-72hpi, the integrity of the plasma membrane and cell viability was carried out using propidium iodide (PI) staining (2,5µg/ml per well) for 15 min. The cells were analyzed by flow cytometry using the FACScalibur and FlowJo software.

### Endothelial Permeability Assay

HBMECs were cultured onto transwell insert (Corning Costar, ME, USA; 0,4 µM membrane), at a concentration of 5x10^4^ cells. The cells were mock treated or infected with DENV-2 (MOI of 1), in the presence or absence of ROS inhibitors, as described. Staurosporine (10 µM; Sigma-Aldrich) was used as a positive control. Cell confluence was monitored before infection and during all the experiment by measuring the transendothelial electrical resistance (TEER), using a Voltohmmeter (Millicell ERS-2), as previously described (30). To calculate the TEER (reported as ‘Ω/cm2’), the membrane resistance itself (without cells) was considered as blank, and the obtained TEER value was subtracted from the resistance value obtained in each experimental conditions; also, the resistance was considered inversely proportional to the area of the membrane. All the experiments were started when a high resistance (> 80 Ω/cm^2^) was reached (31, 32). Endothelial permeability was further evaluated by measuring extravasation of FITC-conjugated BSA through the culture. After 72 hpi, the culture supernatant was removed and a solution of BSA-FITC was added for 30 minutes. BSA extravasation to the lower transwell chamber was quantified using spectrophotometer SpectraMAX i3 (Molecular Devices, Lagerhausstrasse, Austria). The Permeability Coefficient (Pd) of albumin was calculated as described previously (30).

### Analysis of Cytokine Production by ELISA and qRT-PCR

HBMECs were mock-treated or infected with DENV-2, in the presence or absence of NAC, apocynin or mitoTEMPO, and cytokine production was evaluated at 48 hpi, as determined elsewhere (9). The supernatants were harvested and the concentration of secreted CCL5 was determined using the ELISA Development Kit (PeproTech), whereas IL-6 and IL-8 levels were determined using ELISA Ready-SET-Go! (eBiosciences), according to manufacturer’s instructions. The expression of IFN-β mRNA was evaluated in the cell lysates by qRT-PCR. Briefly, RNA was isolated using TRIZOL reagent, and cDNA synthesis was performed using the High-Capacity cDNA Archive Kit (Life Technologies), following the manufacturer’s recommendations. The cDNA was subjected to real-time PCR using Power SYBR Green PCR master mix reagent (Thermo Fisher Scientific Inc.), with the following primers: IFN-β sense: 5’-TAG CAC TGG CTG GAA TGA GA-3′; IFN-b antisense: 5′-TCC TTG GCC TTC AGG TAA TG-3’. GAPDH expression was measured as control gene, using the primers: GAPDH sense 5′-GTG GAC CTG ACC TGC CGT CT-3′, and GAPDH antisense 5′-GGA GGA GTG GGT GTC GCT GT-3′. The reactions were carried out in a StepOnePlus real-time PCR system (Thermo Fisher Scientific Inc.). The comparative CT method (ΔΔCt) (33) was used to quantify gene expression levels.

### Statistical Analysis

Data were analyzed using the GraphPad Prism software (GraphPad Software, San Diego, CA, USA). Comparisons among every two groups were performed by t-test, and two way ANOVA followed by Dunnett’s multiple comparison test were used when different time points were considered; p < 0.05 were considered statistically significant.

## Results

### Infection of HBMEC With DENV-2 Induces ROS Production Through Different Pathways, Which Modulate Virus Replication

Our group demonstrated that infection of HBMEC with DENV-2 induced RIG-I expression, cytokine production, and cell death (9, 12). Since NOX-mediated ROS production has been associated to cellular activation and death upon DENV infection in other cell models (14), here we investigated whether altered HBMEC biology could also be a consequence of DENV-induced oxidative stress.

**Figure 1:**
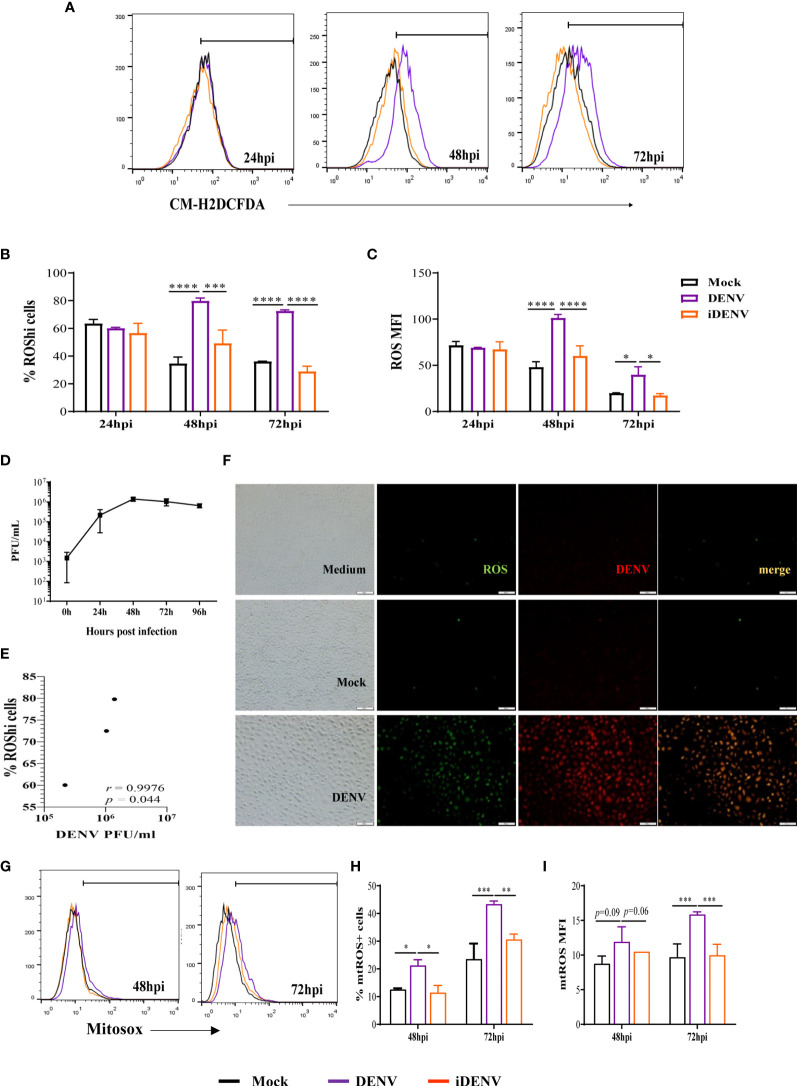
Infection of human brain endothelial cells (HBMEC) with DENV-2 induces ROS production. HBMECs were mock-treated or inoculated with infectious (DENV) or UV-inactivated DENV-2 (iDENV), at an MOI of 1, for the indicated time points. **(A–C)** After 24, 48 and 72hpi, the cells were incubated with CM-H2DCFDA and ROS production was analyzed flow cytometry. A representative histogram overlay is depicted in **(A)** the medians of the frequency of cells producing increased ROS (%ROShi), and the level of ROS production (MFI) obtained from five independent experiments are showed in **(B, C)**. **(D)** The concentration of released infectious particles was measured at the same time points by plaque assay and **(E)** the correlation between DENV-2 titer (PFU/ml) and the frequency of ROS producing cells was analyzed. **(F)** At 72hpi the cells were incubated with anti-DENV antibody and with CM-H2DCFDA probe and analyzed by immunofluorescence. **(G–I)** After 48 and 72hpi, the cells were incubated with MitoSox probe and mtROS production was analyzed flow cytometry. Representative histograms are depicted in **(G)** and the medians of the frequency of cells producing mtROS (%mtROS+ cells), and the level of mtROS production (MFI) obtained from four independent experiments are showed in **(H, I)** *Represents p ≤ 0.05; **p ≤ 0.01; ***p ≤ 0.001; ****p ≤ 0.0001.

HBMECs were infected with DENV-2, at a MOI of 1, and stained with CM-H2DCFDA probe at different time points until 72 hours post infection (hpi). DENV infection stimulated ROS production at 48 and 72 hpi, in comparison to mock-treated cells, which was evidenced by a significant increase in the frequency of cells producing higher ROS levels (% ROShi cells) and in the overall level of intracellular ROS in the culture (MFI) (**Figures 1A–C**). UV-inactivated virus (iDENV) did not affect ROS levels. Also, ROS production was not observed at earlier time points (**Figures S1A, B**), probably reflecting the need for virus replication cycles to amplify the stimulatory signal. As a control, HBMECs were cultured with heme (34), which resulted in increased ROS production after 24h, indicating that there was no intrinsic impairment of the cultures at this time point (**Figures S1C, D**). We evaluated the concentration of released infectious particles in the cultures (PFU) (**Figure 1D**) and observed that ROS levels positively correlated with virus titer overtime (**Figure 1E**). This data was corroborated by immunofluorescence analyses, showing that the majority of DENV-2-infected cells were generating ROS (**Figure 1F**).

In another set of experiments, HBMECs were stained with MitoSox probe to specifically investigate mitochondrial-derived ROS. Increased mtROS were also detected in DENV-infected cells from 48hpi, with a significant enhancement at 72hpi (**Figures 1G–I**).

**Figure 2:**
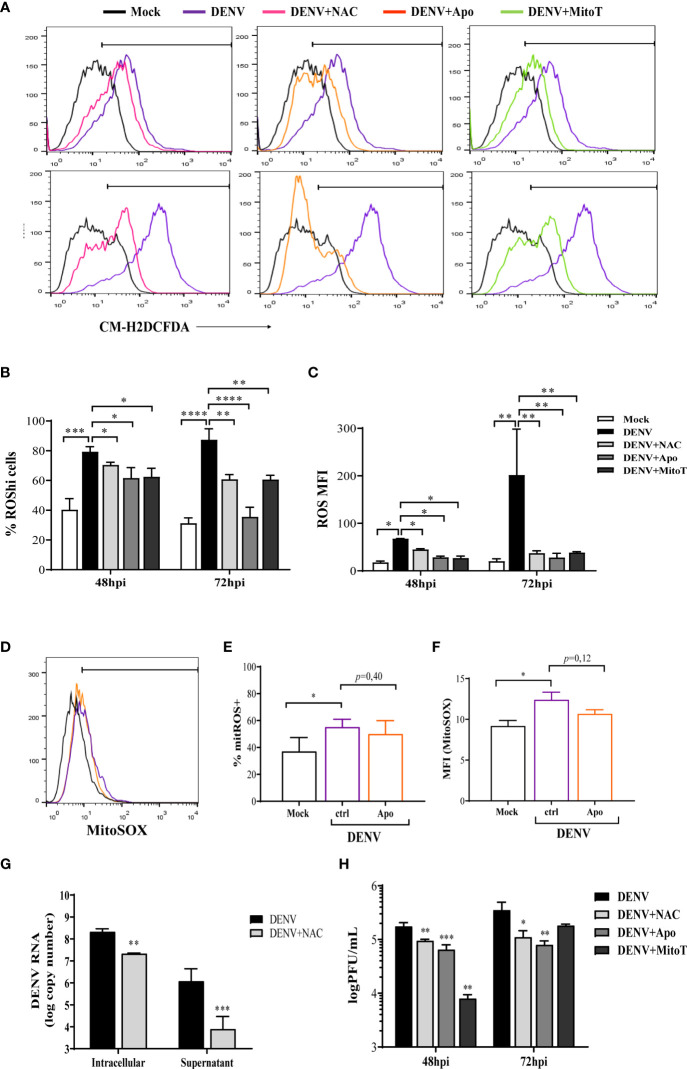
DENV-induced ROS by different intracellular sources modulate virus replication. HBMECs were mock treated or infected with DENV-2, in the presence or absence of N-acetyl-L-cysteine (NAC), apocynin (Apo), or mitoTEMPO (MitoT). **(A–C)** At the indicated time points, the cells were incubated with CM-H2DCFDA probe and the analysis of ROS production was performed by flow cytometry. A representative histogram overlay is shown in **(A)**, the medians of the frequency of cells producing increased ROS (%ROShi), and the level of ROS production (MFI) obtained from four independent experiments are showed in **(B, C)**. **(D–F)** Mock-treated or DENV-infected HBMECs were cultured in the presence or absence of apocynin (Apo) and mtROS production was evaluated by MitoSOX staining and flow cytometry analysis. A representative histogram overlay is shown in **(D)**, the medians of the frequency of cells producing ROS (%ROS), and the level of ROS production (MFI) obtained from two independent experiments are showed in **(E, F)**. **(G–H)** Mock-treated or DENV-infected HBMECs were cultured in the presence or absence of the indicated ROS inhibitors. The concentration of intracellular and released virus RNA were measured by qRT-PCR **(G)** and concentration of released virus particles was evaluated by plaque assay **(H)** data is representative of three independent experiments. *Represents p ≤ 0.05; **p ≤ 0.01; ***p ≤ 0.001; ****p ≤ 0.0001.

To identify the ROS-inducing pathway, HBMECs were infected in the presence or absence of apocynin or mitoTEMPO, to respectively inhibit NOX or scavenge mtROS. The antioxidant N-acetyl-L-cystein (NAC) was used as a control. Decreased ROS levels were observed when HBMECs were cultured with either apocynin or mitoTEMPO, indicating that cytoplasmic and mitochondrial sources are important for ROS generation induced upon DENV-2 infection (**Figures 2A–C**). Viability assays using the drugs alone confirmed that none of the inhibitors were cytotoxic at the concentrations and time points evaluated, supporting their use in the system (**Figure S2**). In addition, since apocynin may function as a ROS scavenger and not as a specific NOX inhibitor in some systems (35), we evaluated whether it could be in fact inhibiting mtROS. The addition of apocynin did not affect MitoSox staining, corroborating with the hypothesis that DENV infection induces ROS generation by different sources in HBMECs (**Figures 2D–F**).

Importantly, ROS scavenging by NAC resulted in diminished intracellular and released virus RNA (**Figure 2G**). Furthermore, addition of NAC, apocynin or mitoTEMPO to the cultures decreased the concentration of released DENV-2 infectious particles, indicating that DENV infection induces the production of ROS, which further contribute to virus replication (**Figure 2H**). Interestingly, inhibition of released virus RNA and infectious particles were more pronounced, in comparison to intracellular RNA reduction, suggesting that ROS may affect later steps of DENV biosynthetic cycle, delaying virus replication.

### DENV-2 Infection of HBMEC Affects Mitochondrial Bioenergetics and Membrane Potential

**Figure 3:**
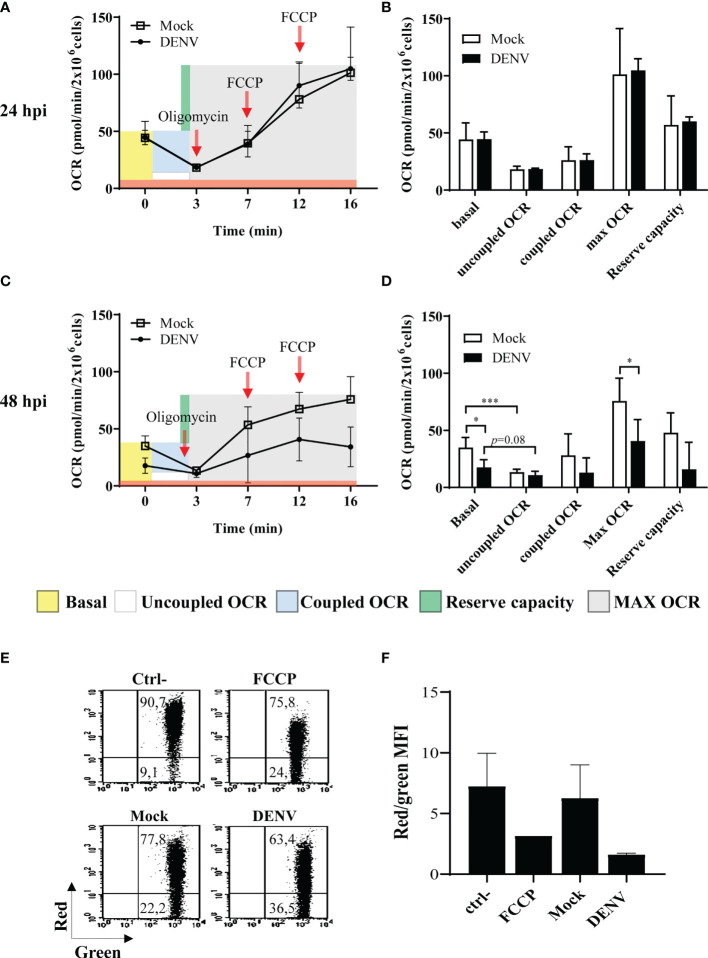
DENV infection impacts mitochondrial function in HBMECs. HBMECs were mock-treated or infected with DENV-2, at a MOI of 1. **(A–D)** At 24 and 48 hpi the oxygen consumption ratio (OCR) was analyzed by high resolution respirometry. OCR in intact cells and after addition of oligomycin and FCCP were sequentially measured **(A, C)** and the obtained values used to calculate the basal respiration, uncoupled OCR (ATP-independent oxygen consumption); coupled OCR (consumption of oxygen dependent on ATP synthase); Max OCR (maximum respiration independent of ATP transport); and the reserve capacity (consumption capacity available during ATP increase) **(B, D)**. Data are represented as mean ± SD of seven independent experiments. *Represents p ≤ 0.05; ***p ≤ 0.001. **(E, F)** Cells were incubated with JC-1 probe and membrane potential was evaluated as the ration red/green fluorescence by flow cytometry. A representative dot plot is shown in **(E)** and the average of the ratio values obtained from three independent experiments are demonstrated in **(F)**.

Virus infection and intracellular virus biosynthesis relies on enhanced energy spent, what may trigger increased respiration. Abnormal respiration may then result in increased electron leakage and generation of mtROS. Indeed, we had demonstrated that infection of hepatic cell lines with DENV resulted in altered mitochondrial bioenergetics and morphology and cell death (25). To evaluate mitochondrial function in DENV-2-infected HBMECs, bioenergetic and membrane potential analyses were performed by high-resolution respirometry and flow cytometry assays. HBMECs were mock-treated or infected with DENV-2 for different periods, and oxygen consumption rate (OCR) was measured after sequentially adding pharmacological modulators of the oxidative phosphorylation. We did not detect any alterations in mitochondrial bioenergetics at 24hpi (**Figures 3A, B**). However, at 48hpi, we observed a significant decrease in the basal OCR in DENV-infected cells, in comparison to the mock treated ones (**Figures 3C, D**). As expected, inhibition of ATP synthase through the addition of oligomycin strongly diminished the OCR in the mock-treated cells (2.7 times, p<0.001). In contrast, oligomycin did not significantly affected the OCRs of the infected cells (1.6 times, p=0.082), which showed similar basal, coupled, and uncoupled values at this time point. In addition, DENV-2-infected cells presented a reduced maximum respiratory capacity evidenced by the lower OCR detected after addition of the proton ionophore FCCP (**Figures 3C, D**). These data indicate that DENV induced mitochondrial membrane leak, detected by diminished basal OCR, which could not be further affected by the modulators of oxidative phosphorylation. In addition, cellular staining with JC1 dye indicated mitochondrial depolarization after 48hpi (**Figures 3E, F**). Taken together, these findings suggest that DENV-2 impairs mitochondrial function and affects membrane potential, resulting in the increased mtROS, which was mostly detected at later time points upon infection.

### DENV-2-Induced ROS Production Promotes Cell Death and Endothelial Permeability

**Figure 4:**
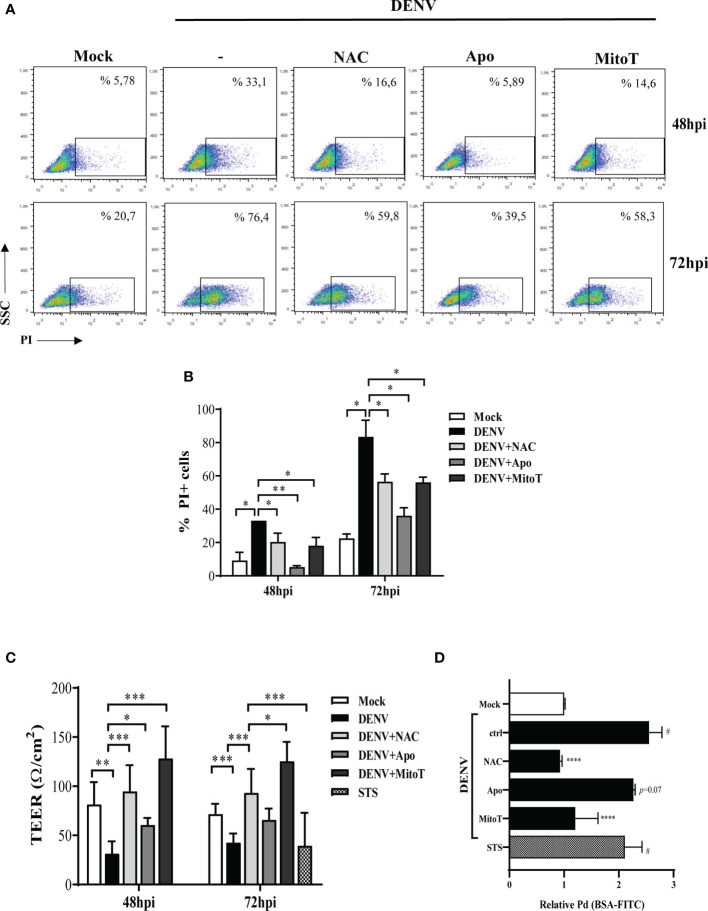
ROS inhibition reduced DENV-induces HBMEC death and partially recover endothelial permeability *in vitro*. HBMECs were mock-treated or infected with DENV-2 for 48 or 72h, in the presence or absence of NAC, apocynin (Apo) or MitoTEMPO (MitoT). **(A, B)** The cells were incubated with propidium iodide (PI) and cell death was evaluated by flow cytometry. A representative dot blot is shown in **(A)** and the bar graph **(B)** demonstrates the average and SD of the frequency of PI^+^ cells (%PI+ cells) obtained from three independent experiments. **(C, D)** HBMECs were cultured onto transwell insert plates and infected with DENV-2, in the presence or absence of NAC, apocynin (Apo) or MitoTEMPO (MitoT). Mock and staurosporin (STS) were used as negative and positive controls, respectively. **(C)** At 48 and 72hpi, transendothelial electrical resistance (TEER) was measured using a volthmeter. **(D)** After 72hpi, the cells were incubated with FITC-conjugated BSA for 1 hour, and the amount of extravasated albumin was measured by spectrophotometry. The permeability coefficient (Pd) was calculated and normalized in relation to cells cultured in medium only. Data represent the mean and SD obtained from four independent experiments. *p ≤ 0.05; **p ≤ 0.01; ***p ≤ 0.001; ****p ≤ 0.0001 in relation to ctrl; ^#^p ≤ 0.0001 in relation to mock.

Since DENV infection impacted HBMEC survival (9), and given that endothelial cell death would affect the endothelium permeability, we investigated whether ROS generation was also involved in these events. Analysis of propidium iodide staining at 48 and 72hpi demonstrated that NAC, apocynin and mitoTEMPO partially reduced HBMECs death induced by DENV-2 infection (**Figures 4A, B**).

We then evaluated whether endothelial permeability induced by DENV-2 would also be restored by reducing ROS accumulation. HBMECs were seed onto transwell inserts and infected with DENV-2, with or without NAC, apocynin or mitoTEMPO, and transendothelial electrical resistance (TEER) was measured at 48 and 72hpi. At 72hpi, culture permeability was also accessed by measuring the extravasation FITC-conjugated BSA through the transwell membrane. Mock and staurosporin (STS) were used as negative and positive controls, respectively. As expected, DENV-2 infection promoted a decrease in the TEER, associated to increased extravasation of BSA-FITC to the lower transwell compartment (**Figures 4C, D**), demonstrating that DENV-2 induces permeability in this cell model. HBMEC permeability was completely rescued when the cells were treated with NAC or mitoTEMPO. Addition of apocynin resulted in increased TEER but did not significantly protect the monolayer from BSA extravasation, suggesting that mtROS might be a major mediator in DENV-2-induced endothelial permeability.

To investigate whether these events were specific to endothelial cells, we evaluated ROS production, virus replication, and cell viability after infection of primary human macrophages with DENV-2. DENV-infected macrophages also showed increased total and mtROS production as evidenced by staining with CM-H2DCFDA or MitoSox probes (**Figures S3A, B**). As observed for HBMEC, macrophage treatment with NAC resulted in diminished virus replication and cellular survival (**Figures S3C, D**), indicating that oxidative stress induced by DENV-2 infection might be essential for fueling virus replication, resulting in cell death.

### Apocynin-Modulated ROS, But Not mtROS, Is Essential for the Secretion of Inflammatory Cytokines Induced by DENV-2 Infection of HBMECs

**Figure 5:**
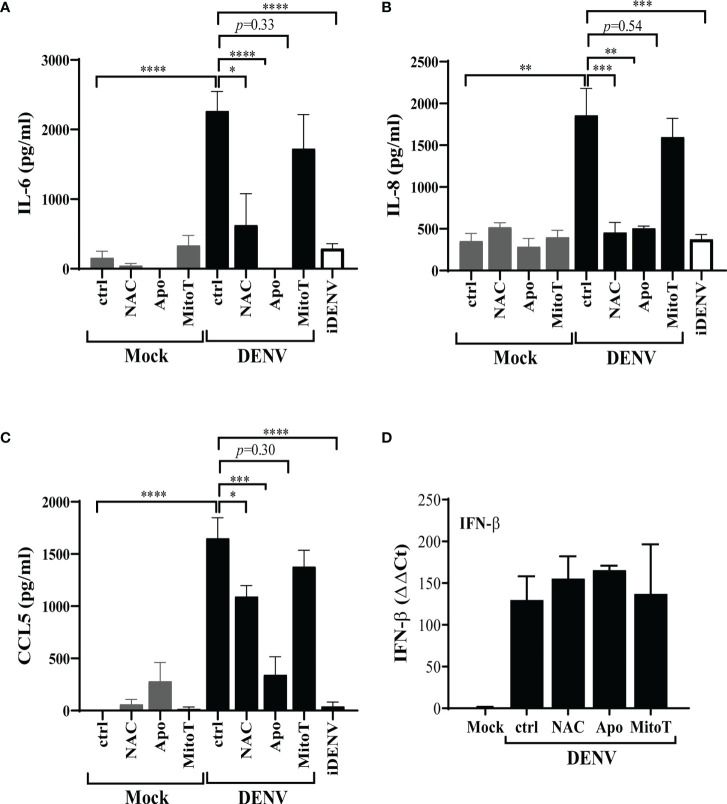
Apocynin-inhibited ROS is essential for the secretion of inflammatory cytokines induced by DENV infection of HBMECs. HBMECs were mock treated or infected with DENV-2 for 48h, in the presence or absence of NAC, apocynin (Apo) or mitoTEMPO (MitoT). **(A–C)** The supernatants were harvested and the concentration of IL-6 **(A)**, IL-8 **(B)**, and CCL5 **(C)** were measured by ELISA. **(D)** The expression of IFN-β mRNA was measured in the cell lysates by quantitative real time PCR. Data represent the mean and SD obtained from three independent experiments. *p ≤ 0.05; **p ≤ 0.01; ***p ≤ 0.001; ****p ≤ 0.0001.

Given that HBMEC infection with DENV-2 promoted cellular activation (9) and since accumulation of intracellular ROS had been previously proposed to stimulate immune responses in other human cell types (14), we investigated whether this stress response could also contribute to HBMEC activation. HBMECs were infected, in the presence or absence of ROS inhibitors, and IFN-β expression and IL-6, IL-8 and CCL5 secretion were measured by qRT-PCR and ELISA, respectively. Cytokine secretion was significantly inhibited by NAC and apocynin, but not by mitoTEMPO (**Figures 5A–C**), indicating that other ROS sources, but not mtROS, contributes to signal transduction associated to IL-6 and chemokine release. Surprisingly, increased IFN-β expression induced by DENV-2 infection was not modulated by any inhibitors (**Figure 5D**).

## Discussion

Dengue severity correlates with exacerbated inflammation, cytokine storm, vascular hyperpermeability, and plasma leakage (2, 3, 36). Enhanced circulation of inflammatory cytokines has been assumed to be the major cause of vascular lesion (4–6). However, virus replication in endothelial cells may directly affect endothelium integrity or synergize with the inflammatory mediators, further contributing to dengue-mediated vascular dysfunction. In the present study we demonstrated that infection of HBMEC by DENV results in increased generation of ROS, which in turn modulates virus replication, cell death and cellular activation.

Evidence of oxidative stress has been reported in patients and *in vivo* experimental models. Plasma obtained from dengue patients showed higher levels of lipid oxidation and increased ratio of protein carbonylation (PCOs) in relation to protein-bound sulphydryl (PBSH) group, which are indicatives of protein oxidation and decreased plasma antioxidants (37). Importantly, protein and lipid alterations were detected early after the symptoms onset and positively correlated with dengue severity and cytokine storm (38).

Using a mouse model of dengue infection, Yen and collaborators showed that iNOS- and 47phox-deficient mice were partially protected from hemorrhage development (22). In this model, DENV antigens were detected in hemorrhagic tissues in association with CD31+ endothelial cells, which also showed an apoptotic phenotype, potentiated by TNFα. In addition, *in vitro* infection of HUVEC with DENV-2 resulted in increased iNOS and NOX activity, which appeared to be related to DENV-induced apoptosis. We have previously demonstrated that HBMECs are productively infected by DENV, resulting in prompt cellular activation and late cell death (9), what might be associated to the increased vascular permeability. In this HBMEC model, we have now showed that DENV replication stimulated ROS generation, which depended on virus replication. It is important to notice, however, that most of the probes available to measure ROS may eventually react with other species such reactive nitrogen species (RNS) (39). Although we cannot discard that DENV also increased nitric oxide production, DCF staining was reduced by apocynin and mitoTEMPO inhibitors, supporting ROS production by DENV-infected HBMEC. Also, ROS inhibition reduced cell death, corroborating the previous reported data regarding HUVEC infection.

Infection of HBMECs, however, also induced mtROS, probably as a consequence of mitochondrial dysfunction. Altered mitochondrial bioenergetics and depolarization of mitochondrial membrane were clearly observed after 48hpi, when increased mtROS was detected with additional increase afterwards. Inhibition of mtROS also reduced cell death, suggesting that DENV-induced mitochondrial stress further contribute to HBMEC death.

Several pathological conditions have been associated to abnormal mtROS generation, due to inefficient production of ATP, altered NADH/NAD+ ratio in the matrix, or inner membrane depolarization, promoting an unbalanced escape of electrons from complex I and III (24, 40, 41). Increased ROS may then act in a feedback loop, inducing the depolarization of membrane potential, and impairment of oxidative metabolism, potentiating mitochondrial damage (42, 43). Interaction of viral proteins with mitochondrial membranes, leading to depolarization and increased permeability, has been largely described, and leakage of mitochondrial content was associated to apoptosis (44–46). Additionally, recruitment and activation of mitochondrial antiviral-signaling protein (MAVS) triggered by cytoplasmic sensing of virus RNA was also associated to cell death in *in vitro* infection models (47).

We had demonstrated that infection of hepatocytes with DENV as well as of neuroblastoma cells with Sindbis virus induced altered mitochondrial bionenergetics (25, 48). These events probably reflect additional energy demands during virus replication with disruption of the energetic mitochondrial flux. Increased ATP flux might be necessary for efficient virus replication, whereas sustained stress resulted in cell death (48).

Despite possible antiviral effects of oxidative stress responses (49, 50), we observed that ROS inhibition reduced DENV replication in HBMEC. Different mechanisms might explain this phenomenon. Like our model, infection of A549 cells with Respiratory Syncytial Virus (RSV) altered mitochondrial bioenergetics, evidenced by lower basal OCR and decreased maximal respiratory capacity. Enhanced ROS production was also detected and inhibition of ROS dampened virus replication (51, 52). Taken together with other morphological and functional alterations detected in the mitochondria, one can suggest that mitochondrial components might be coopted by viruses favoring their replication. Accordingly, HBMEC treatment with mitoTEMPO decreased the production of DENV infectious particles, indicating that mitochondrial dysfunction is also associated with viral replication in this system. Decreased effect of mitoTEMPO at later time points may indicate that mtROS scavenging is hindering, but not preventing virus replication. Supplementation of the cell culture with ROS inhibitors overtime could foster their effect. Still, more than 50% inhibition in the virus titers were detected at 72hpi, even by adding mitoTEMPO only at the beginning of the culture.

Increased ROS may also trigger autophagy, and subversion of autophagy machinery has been demonstrated to benefit viral replication in different infection models, including dengue (53, 54). Infection of monocytes with different flaviviruses induced autophagy and this event was important to protect the cells from other stress responses and early cell death. Early inhibition of cellular stress contributed to virus replication (55). Accordingly, increased LC3/LC3II conversion and accumulation of p62 was observed in HBMECs infected with at 24hpi, but not at later time points (data not shown), suggesting that autophagy may be an earlier event conferring cell protection. These events will be further investigated. On the other hand, ROS-mediated cell death phenotype started to be detected at 48hpi, being significantly increased at 72hpi, suggesting that apoptotic or necroptic events are later triggered probably as a resulted of sustained ROS production.

We could only detect significant ROS enhancement after 48hpi, what indicates that sequential virus replication cycles might be necessary to amplify the response. In fact, we previously observed that HBMEC infection with a MOI of 1 resulted in about 30% of infected cells at early time points (9). Alternatively, earlier oxidative stress could be impaired by stimulation of antioxidant responses. DENV infection of monocytes derived dendritic cells (mo-DC) induced NOX-mediated late ROS response, which was also associated to cell death in that model (14). It was also showed that Nrf2 antioxidant was increased at earlier time points, but it was later degraded due to action of NS2BNS3 viral proteins, allowing ROS accumulation (16). Nrf2 depletion resulted in increased frequency of DENV infected cells, indicating that ROS was also important for virus replication in those cells.

Besides fueling virus replication, NOX-dependent ROS production in Mo-DC resulted in activation of inflammatory signals, with the production of IFN-β, IL-1β and CCL5 (14, 16). In our model, treatment of DENV-infected HBMEC with apocynin inhibited CCL5, IL-6, and IL-8 secretion, suggesting that NOX-derived ROS may also take part in vascular inflammation induced by the virus. It is important to notice, however, that apocynin may not function as a *bona fide* NOX inhibitor in endothelial cells. Heumuller and colleagues described that porcine aortic endothelial cells (PAEC) might not express myeloperoxidase and failed to form apocynin dimers, which would be essential to its activation and NOX inhibition (35). In these cells, apocynin mostly functioned as a peroxidase scavenger. In another set of studies, using a HUVEC-derived cell free system, it was demonstrated that addition of peroxidase to the system was indeed important to form apocynin dimer and that those dimers induced a prompt and almost complete inhibition of O2- production. Still, apocynin monomers also resulted in decreased O2- production, although after a lagtime (56). It is important to point that previous studies addressing multiple effects of oxidative stress specifically in HBMECs have reported that apocynin reduced the activation of NADPH oxidases or, at least, NOX-dependent cellular dysfunction (57–59), highlighting the complexity and diversity of vascular endothelial models. Importantly, in DENV-infected HBMEC model it was clearly demonstrated that apocynin-inhibited ROS, but not mtROS, was a major contributor to the secretion of inflammatory cytokines and chemokines. Therefore, although we had not fully elucidated the source of non mtROS, it is worth to mention that inhibition of ROS by apocynin might be a potential strategy to control DENV-induced inflammation. It was previously demonstrated that cytoplasmic ROS may induce MAVS oligomerization, potentiating RIG-I-MAVS signaling pathway, independent of virus RNA sensing (60). Interestingly, IFN-β expression was not affected by ROS inhibition, suggesting that ROS may differentially impact NF-κB and IRF signaling pathways, what should be further investigated.

Cell death and NF-kB inflammatory signaling pathways were also detected after infection of neuroblastoma cells with DENV (61). Given that the endothelial cells used in this study are a representative model of *in vitro* blood brain barrier (BBB), we can speculate that when DENV achieves the BBB and the central nervous system, ROS production might contribute to virus invasion and neuroinflammation. Finally, DENV-induced ROS was important for increased endothelial permeability and mtROS appeared to be the major pathway, although inhibition of NOX activation also increased the TEER.

**Figure 6:**
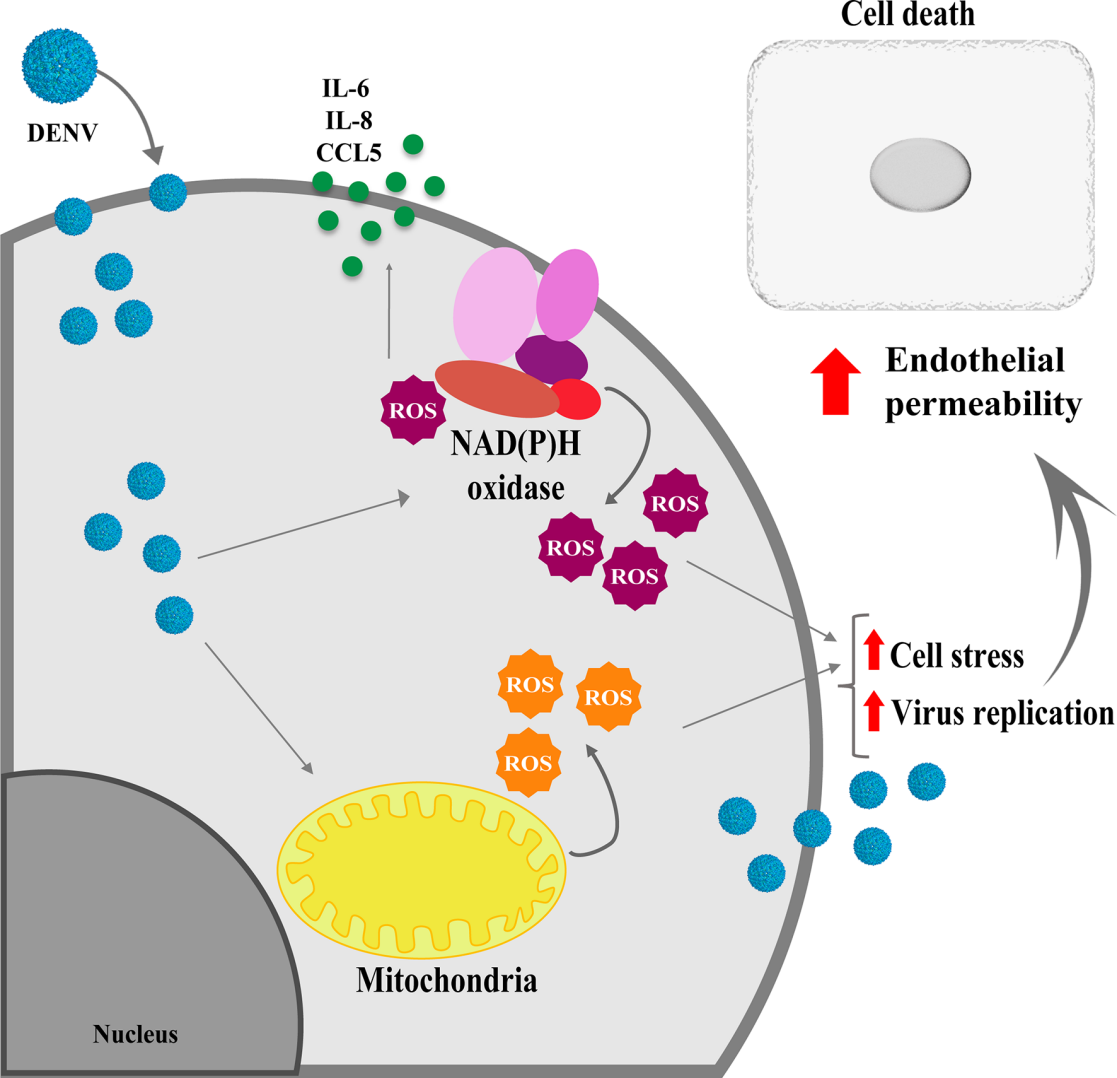
Schematical representation showing the effects of ROS induced by DENV infection in HBMECs. Infection of endothelial cells by DENV-2 impact the mitochondrial physiology, leading to ROS production, which will fuel virus replication and induce cell death, contributing to endothelial permeability. In addition, activation of other intracellular sources, such as NOX enzymes further enhance ROS production, which will be essential to increase secretion of chemokine and inflammatory cytokines.

Taken together our data indicate that altered metabolism triggered by DENV replication results in ROS production from different cell sources, which is important for virus replication, endothelial activation, and increased permeability (**Figure 6**). Further studies addressing the effect of antioxidants *in vivo* may contribute to avoid vascular permeability, inflammation and neuroinvasion upon DENV infection.

## Data Availability Statement

The raw data supporting the conclusions of this article will be made available by the authors, without undue reservation.

## Ethics Statement

The studies involving human participants were reviewed and approved by Experimental Ethics Committee of Universidade Federal do Rio de Janeiro. Written informed consent for participation was not required for this study in accordance with the national legislation and the institutional requirements.

## Author Contributions

Conceptualization, LA. Data curation, LM and EP. Formal analysis, AP, MB, and LA. Funding acquisition, AP, MB, and LA. Investigation, LM, EP, MP, YM, AP, MB, and LA. Methodology, LM, LRPC, LSC, AP, MB, and LA. Project administration, LA. Resources, LRPC, AP, MB, and LA. Supervision, LA. Writing – original draft, LM, EP, and LA. Writing – review & editing, AP, MB, and LA.

## Funding

This work was supported by Coordination for the Improvement of Higher Education Personnel (CAPES); Brazilian National Council for Scientific and Technological Development (CNPq; #405323/2016-6; #310867/2018-5); Carlos Chagas Filho Research Support Foundation (FAPERJ; #E-26/201.324/2016; #E-26/210.371/2019, #E-26/201.206/2021; #E-26/010/002999/2014; #E-26/202.877/2018; #E-26/010.02425/2019); Funding Authority for Studies and Projects (FINEP). LM, AP, MB, and LA were recipients of a CNPq fellowship.

## Conflict of Interest

The authors declare that the research was conducted in the absence of any commercial or financial relationships that could be construed as a potential conflict of interest.

## Publisher’s Note

All claims expressed in this article are solely those of the authors and do not necessarily represent those of their affiliated organizations, or those of the publisher, the editors and the reviewers. Any product that may be evaluated in this article, or claim that may be made by its manufacturer, is not guaranteed or endorsed by the publisher.
