# Supplementary material for: Infection of Endothelial Cells by Dengue Virus Induces ROS Production by Different Sources Affecting Virus Replication, Cellular Activation, Death and Vascular Permeability
Source: Front Immunol. 2022 Feb 2;13:810376. doi: 10.3389/fimmu.2022.810376 (PMC8847576; doi:10.3389/fimmu.2022.810376)
Supplement: Supplementary file 1 [file DataSheet_1.pdf]

Supplementary Material

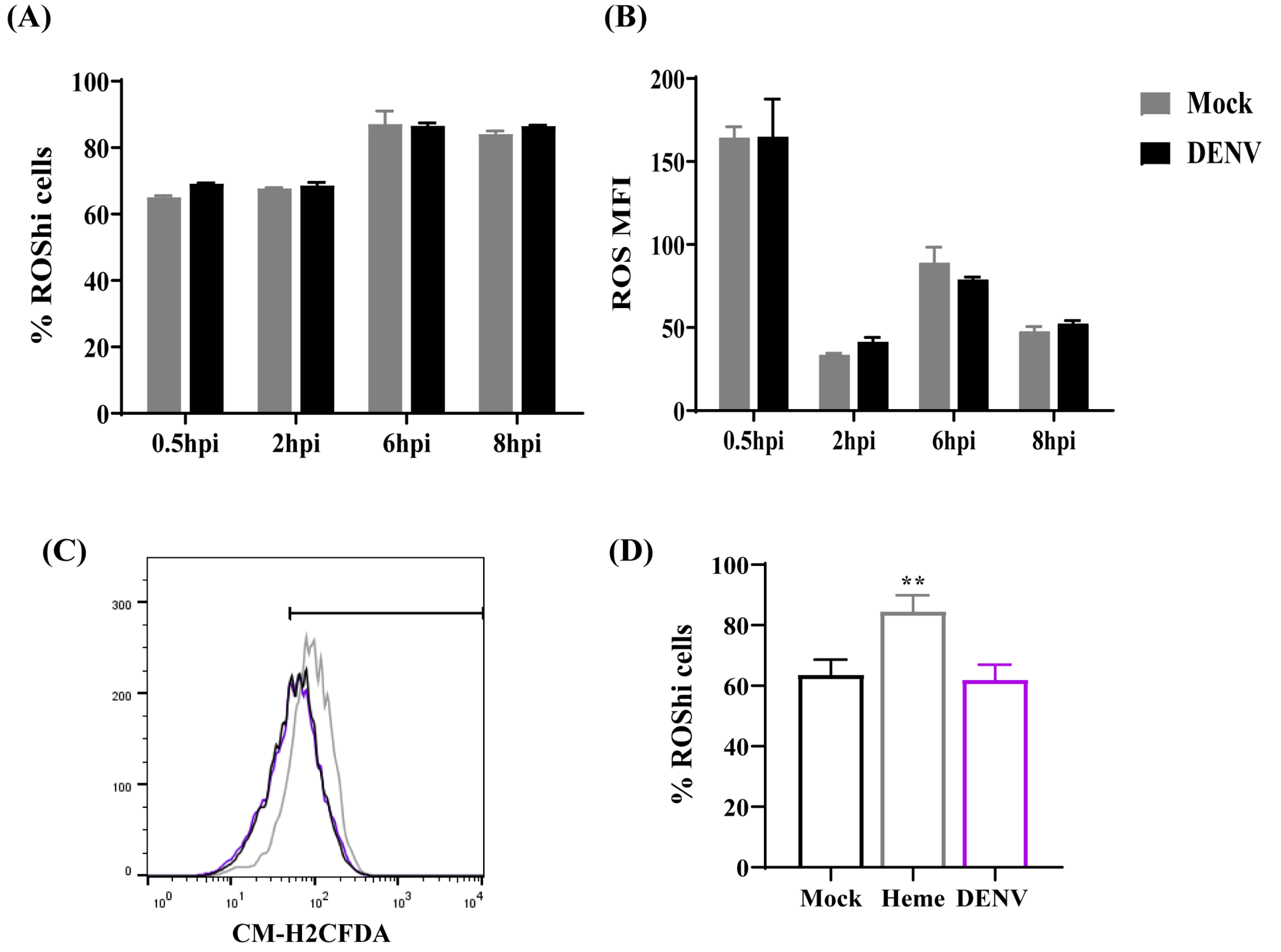

Figure S1. DENV infection does not induce ROS production in the early stages of infection. HBMECs were mock-treated or infected with DENV-2 (MOI 1). **(A-B)** After 0.5, 2, 6 and 8 hpi, the cells were incubated with CM-H2DCFDA probe and the frequency of cells producing increased ROS levels (% ROShi) **(A)** and ROS production level (MFI) **(B)** were analyzed by flow cytometry. Data is representative of three independent experiments. **(C-D)** HBMECs were cultures with mock, DENV-2 or heme and, after 24h, the cells were stained with CM-H2DCFDA. A reperesentative histogram and the median average of two independent experiments is shown in **(C)** and **(D)**; **represents $p \leq 0.01$ in relation to mock.

Figure S2

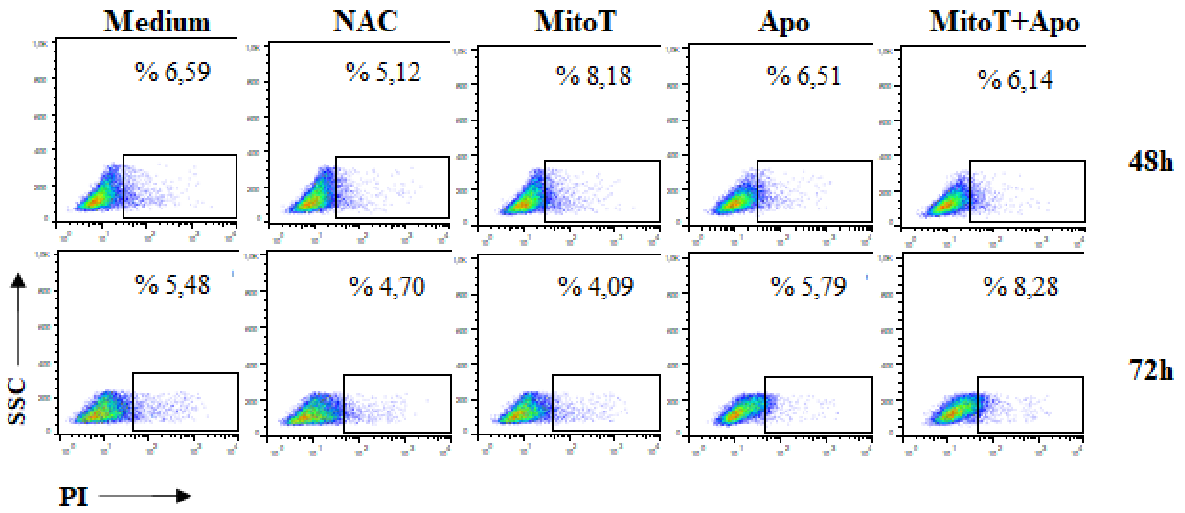

Supplementary Figure 2. Cytotoxic analysis of ROS inhibitors. HBMECs were cultured with N-acetyl-L-cysteine (NAC; 1mM), apocynin (Apo; 1mM), or mitoTEMPO (MitoT; 50µM). After 48 or 72hpi, the cells were stained with propidium iodide (PI) and analyzed by flow cytometry; the frequency of PI positive cells (%) is showed in the inserts.

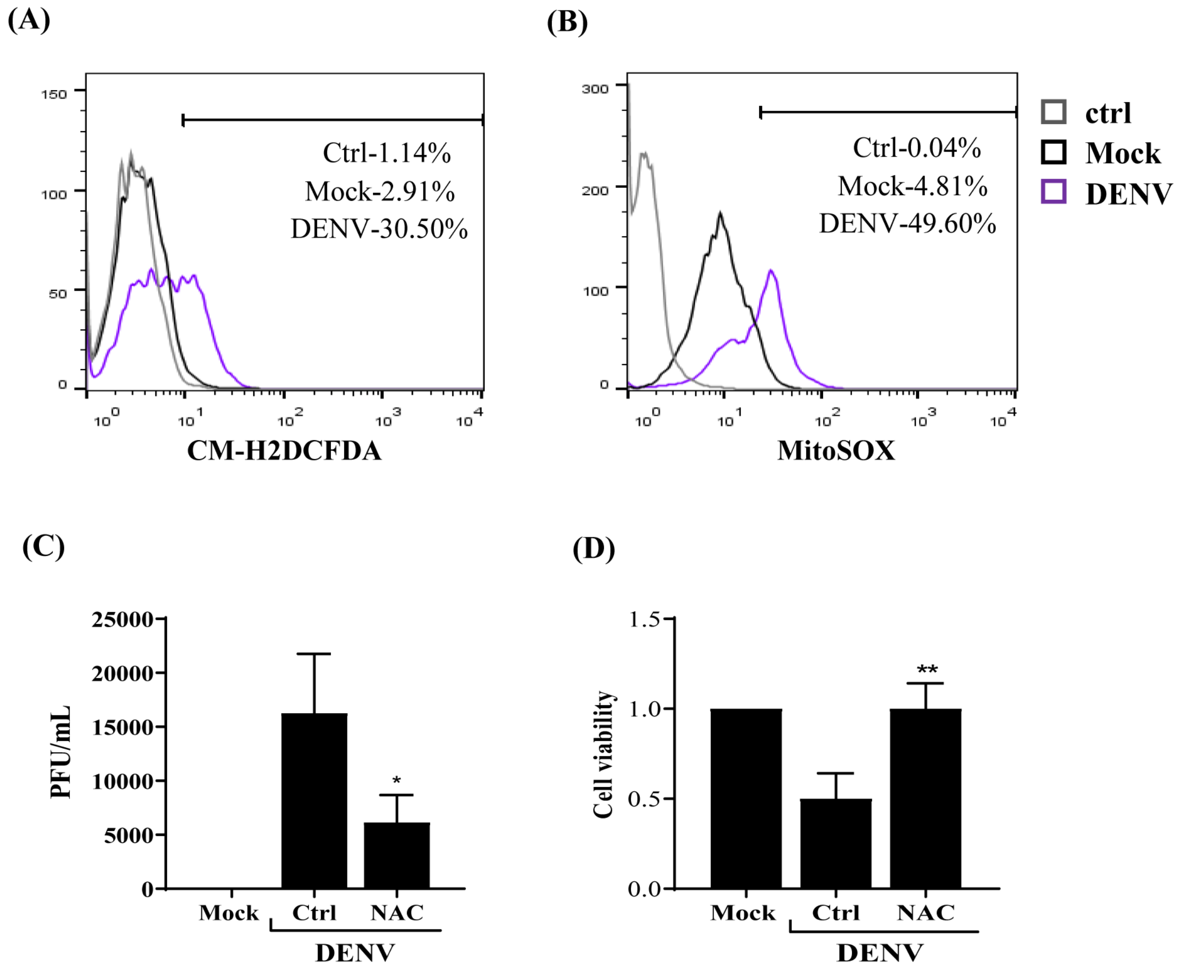

Figure S3. DENV-induced ROS production modulates virus replication and cell viability in human primary macrophages. Human macrophages were mock-treated or infected with DENV-2 for 48h, in the presence or absence of N-acetyl-L-cysteine (NAC). **(A-B)** The cells were incubated with CM-H2DCFDA **(A)** or MitoSox **(B)** probes and the analysis of ROS production was performed by flow cytometry. **C)** The titer of infectious particles released in the culture medium was evaluated by plaque assay. **D)** Cell viability was accessed by MTT assay. Data are representative of three independent experiments; * represents $p \leq 0.05$; ** $p \leq 0.01$.
